# Barriers to and enhancement of the utilization of digital mental health interventions in low-resource settings: Perceptions of young people in Uganda

**DOI:** 10.1177/20552076251321698

**Published:** 2025-02-16

**Authors:** Erisa Sabakaki Mwaka, Datsun Bazzeketa, Joy Mirembe, Reagan D. Emoru, Adelline Twimukye, Apollo Kivumbi

**Affiliations:** 1 School of Biomedical Sciences, 58589 College of Health Sciences, Makerere University, Kampala, Uganda; 2 Faculty of Science and Technology, 438586 International University of East Africa, Kampala, Kampala, Uganda; 3 Faculty of Science and Computing, 127816 Ndejje University, Kampala, Kampala, Uganda; 4 242941 Infectious Diseases Institute, Makerere University, Kampala, Central, Uganda; 5 37412 Dornsife School of Public Health, Drexel University, Philadelphia, PA, USA

**Keywords:** Young people, digital mental health, mental health, access, equity

## Abstract

**Introduction:**

Digital mental health (DMH) enhances access to healthcare, particularly in low- and middle-income countries where investment in mental healthcare is low. However, utilization among young people (YP) is low. This study aimed to explore YP's perceptions of the barriers to the using of DMH interventions in low-resource settings.

**Methods:**

A qualitative descriptive approach was used. Six face-to-face focus group discussions were conducted with 50 YP from nine universities in Uganda. The median age was 24 years (range 21–25 years) and respondents were drawn from diverse academic programmes with the majority being medical students (54%). A thematic approach was used to interpret the results.

**Results:**

Three themes were identified from the data including perceptions of using DMH services, the perceived barriers to utilization, and suggestions for enhancement of DMH for YP in low-resource settings. Most respondents had a positive attitude towards DMH. The perceived barriers to utilization of DMH included the fear of stigma, affordability, inequitable access, privacy and confidentiality concerns, and app-related challenges. Access and use of DMH can be enhanced through public engagement, creating awareness, enhanced training, and access to affordable DMH interventions.

**Conclusion:**

DMH was deemed important in extending healthcare to YP, particularly in health systems where traditional mental health services are not readily available. However, several factors hinder equitable access to DMH in low-resource settings. There is a need for long-term investment in digital health technologies.

## Introduction

Globally, mental disorders account for 14% of the global burden of disease among young people (YP) aged 10–24 years^1^; yet they remain obscure and under-treated.^2^ Mental health disorders are characterized by unexpected disturbances in an individual's cognition, emotion, and behavioural control, preventing them from functioning effectively.^3^ The most prevalent mental health disorders among YP include depression, anxiety, and behavioural disorders.^2^ Mental disorders that are initiated early in life tend to continue manifest in adulthood.^4,5^ The global onset of the first mental disorder occurs before 14 years in 34.6% of individuals, 18 years in 48.4%, and before age 25 in 62.5%, with a peak/median age at onset of 14.5/18 years across all mental disorders.^6^ Mental disorders are associated with several long-term effects if untreated. For example, people living with mental illness have a poor quality of life^7,8^; are at increased risk of health disorders—stigma, marginalization, and discrimination, especially due to traditional beliefs^9^; excess years lost and excess mortality^10^; higher lost disability-adjusted life years,^11^ and increased risk of suicide, accidental death, and premature mortality.^12^

The SARS-CoV-2 (COVID-19) pandemic had a big impact on the mental health of children and YP mostly due to associated restrictions,^13,14^ loneliness,^15,16^ substance abuse,^17,18^ uncertainty about work futures,^19^ food insecurity, and violence.^20^ Active Minds, a non-profit organization promoting mental health for young adults in the USA, in a survey, reported that one in every five students experienced significantly worsened mental health due to COVID-19, 80% indicated that the COVID-19 pandemic impacted their mental health, and 55% reported that they did not know where to seek for help.^21^ Another study from South Africa reported anxiety and depression in 45.6 and 35% of 5074 university students respectively.^22^

Mental disorders affect a substantial proportion of the population in Uganda. The prevalence of mental illness among children and adults in Uganda is higher than the global average of 11.6%,^23^ and is estimated to be between 24.2%^24^ to 32.0%.^25^ The prevalence of any mental disorder in children in Uganda is estimated at 22.9%, with major depressive, anxiety, and post-traumatic stress disorders being the most prevalent.^24^

It is important to note that Uganda is home to one of the biggest refugee populations in the world, with most of them running away from protracted war conflicts in their home countries. Uganda also has a history of a civil war that ravaged the northern region of the country for more than 20 years. It has been proven that populations in such conflict areas exhibit an increased prevalence of mental disorders.^26,27^ Further, there was an exponential increase in mental health problems among children YP in Uganda during the COVID-19 pandemic.^18,20,28^ Matovu et al., in a survey involving 2500 adolescent boys and young men aged 10–24 years during the COVID-19 lock-down in Uganda, revealed that 70.3% of respondents reported feeling more nervous, 63% reported feeling sad, 55.7% felt more hopeless, and 1.2% contemplated committing suicide.^29^ It is important to note that in that study, older adolescent boys and young men were more significantly affected.^29^ This increase in common mental health problems led to an increase in the search for solutions. At the time, there was a surge in the use of digital mental health (DMH) systems to reach out to YP and offer much-needed mental healthcare.^30,31^

From the above account, it is evident that there is a dire need for quality mental health care services in Uganda. However, Uganda suffers an acute shortage of mental health service providers and funding for mental health services is inadequate. There are only 53 psychiatrists in Uganda, with each psychiatrist serving almost 1 million people; and most of them are located in urban centres.^24,25^ The low coverage of mental health services within the country and on the African continent during the COVID-19 pandemic provided an opportunity for digital solutions, especially among YP.^32^ Ensuring equitable access to mental health care is critical for the health and well-being of YP because as already noted above, mental disorders persist from a young age. Further, YP are vulnerable to social exclusion, discrimination, the stigma that hinders them from accessing help, educational challenges, risk-taking behaviour, physical health problems, and human rights violations.^2^

The lack of specialized mental health service providers continues to be a barrier to the delivery of mental health care in low- and middle-income countries (LMICs), including Uganda. DMH is seen as a promising route to tackle the treatment gap, but it also raises ethical, legal, and policy challenges. Wykes et al. defined DMH as ‘all technologies that provide treatment and management of mental health problems’.^33^ DMH interventions have the potential to overcome some existing barriers to conventional care and increase access to mental health support services and resources.^34^ Digital health can be delivered through a range of media including mobile phone apps, internet websites, telemedicine, wearable devices, virtual reality products, video games, and self-guided digital health interventions.^32,34,35^ Although the effectiveness of some DMH interventions has been reported to be comparable to conventional mental health services in improving mental health conditions such as depression and anxiety,^36,37^ uptake is low.^38,39^

Studies from sub-Saharan Africa have reported low awareness of DMH.^38,40,41^ They have also reported several hindrances to the uptake of DMI including lack of cultural relevance, high internet costs, privacy concerns, low digital literacy and other technological challenges,^38^ and lack of national digital health frameworks to oversee and manage digital health.^40^ Despite these barriers, there are positive attitudes towards DMH and a high intention to use DMH solutions.^38,42^ Studies have also reported the acceptability and feasibility of using DMH in improving access to mental health and psychosocial services.^31,41^

DMH is a relatively new concept to Uganda, as such, there is a dearth of literature on the potential facilitators and barriers to the adoption of DMH interventions. This study therefore set out to explore YP's perceptions of the barriers to the utilization of DMH interventions, and suggest contextualized solutions to enhancing the adoption of DMH in in low-resource settings.

## Materials and methods

A qualitative descriptive approach was used to collect data on YP's perceptions of the barriers to the utilization of DMH interventions in low-resource settings. A qualitative descriptive approach provides a comprehensive summary and straightforward description of the phenomenon.^43^ In qualitative descriptive studies, the researcher seeks descriptive validity, or an accurate description of the event that most people (including investigators and respondents) observing the same event would agree is accurate; and interpretive validity, or an accurate account of the meanings respondents attributed to events that respondents would agree is accurate.^44^ Qualitative descriptive studies are based on a generic orientation where there is no a priori commitment to any one theoretical view of a phenomenon.^43^ This study had no a priori theoretical orientation, and the results are presented as described by the respondents.

### Study population

The study involved face-to-face focus group discussions (FGDs) with YP from nine public and private universities in Uganda. YP (young adults) were defined as individuals aged 18–25 years according to the Society for Adolescent Health and Medicine.^45^ Only university students, aged 18–25 years, and willing to provide written informed consent were enrolled in the study. Eligible students who had untreated neurocognitive conditions that affect a person's capacity to provide valid informed consent were excluded from the study. This determination was done by AK who was a senior resident of psychiatry. We selected university students because, at the time, there were reports of an increase in mental health disorders among university students (Monitor, 2018). All the students we approached accepted to participate. A Young People's Advisory Group (YPAG) was constituted under the leadership of two dynamic YP to mobilize and recruit participants. In turn, the YPAG was directly supervised by two early career researchers (ECRs). The ECRs were purposively selected based on their expertise in psychiatry and digital innovation for health respectively. A snowballing approach was used with peer referral for participant recruitment. The YPAG identified some eligible YP who in turn invited their peers to participate. We ensured diversity by enrolling students from various undergraduate academic programmes. A total of 50 YP participated in the FGDs, of which a majority were male (31/50, 62%). The median age of respondents was 24 years (range 21–25 years). The majority of respondents were pursuing a Bachelor of Medicine and Bachelor of Surgery (27/50, 54%). Other academic programmes represented included bachelors of Dental Surgery, Veterinary Medicine, Law, Business Administration, Software Engineering, Education, Information Technology, and Marketing. Three quarters (38/50, 76%) were in their final year of study. Almost all respondents (48/50, 96%) owned a smart mobile phone. Forty-three (86%) had ever considered seeking help for a mental health problem.

#### Data collection

Six face-to-face FGDs were conducted between February and August 2022 by a team of five that included the principal investigator (ESM); two ECRs, one of whom had experience in qualitative research methods (AK), and the other in digital innovations (DB); and two YPAG who were fifth-year medical students (JM and RDE). The same team of five conducted all the FGDs to ensure consistency. Before the start of the study, the research team was trained on the protocol to ensure that they internalized and understood the study well. Data were collected using an FGD guide that was developed collaboratively by the Ethics for Mental Health Digital Innovations for Young People in Africa (EMDIYA) network. The EMDIYA network brings together stakeholders and experts in the ethics of DMH, digital innovators, mental health practitioners, and policymakers from five African countries.^46^ The chief aim of the network is to develop a robust framework for responsible and relevant DMH interventions for YP in African countries. The trans-disciplinary network has a group of YP at its core, who co-create all its work and outputs. A more detailed description of the EMDIYA project is available elsewhere.^47^

The FGD guide consisted of open-ended questions that explored YP's awareness; perceptions of the barriers to the utilization of DMH interventions; and suggestions on how to enhance access to DMH interventions by YP in low-resource settings. The FGD guides were piloted on a group of three YP before the full data collection process to ensure that the questions were appropriate and easy to understand. Those who participated in tool-piloting were excluded from the study.

A brief description of the study and details of the set date, time, and venue for the FGD were communicated a week before the discussions by phone. A follow-up call was made the day before the discussion to confirm availability.

All FGDs were conducted at the same location at one of the universities, and comprised eight to nine male and female participants. Only respondents participated in the FGDs. On arrival, the ECRs provided full details of the study and written consent was obtained. The discussions were initiated and moderated by research team members guided by a semi-structured discussion guide with open-ended questions. Open-ended questions allowed easy flow of the discussions and provided participants a platform to share their thoughts, opinions, and experiences on DMH. All FGDs were conducted in English, audio-recorded alongside detailed notetaking, and later transcribed verbatim. After the sixth FGD, no new codes were emerging, therefore, it was deemed that we had attained a saturation point.^48^ Transcripts were cleaned before the analytical process. On average, interviews lasted between 60 and 90 minutes. Debriefing meetings were held by the research team at the end of each interview to check on completeness and review preliminary perspectives that had arisen. All data were securely kept ensuring confidentiality.

#### Data management and analysis

The audio-recorded discussions were organized and transcribed verbatim, and each transcript was typed and saved using a corresponding unique identifier to the audio. Data analysis was conducted continuously throughout the study using a thematic approach.^49,50^ Three authors (ESM, AK, and AT) then developed a codebook and coding framework. A coding framework was designed based on three transcripts manually reviewed and coded to create the initial set of codes developed by three coders. All the transcripts later were imported into NVivo version 12 (QSR International Pty Ltd, 2014) for open coding and codes were checked iteratively among three coders trained in qualitative data analysis to enhance reliability, and a final codebook was developed. We assigned codes to relevant segments of the text, and similar or related codes aggregated to form themes. Final codes and themes were organized using NVivo 12 software (QSR International Pty Ltd, 2014). Themes were generated inductively, by considering the themes that emerged from the data. Then ESM, AK, DB, and AT examined the themes for patterns until consensus was achieved on the final themes after several iterative discussions.

Transcripts were not returned to respondents for checking however, AK and DB read through the transcripts several times and cross-checked with the audio files to ensure that they were accurate. We applied the consolidated criteria for reporting qualitative research (COREQ) checklist for qualitative studies to the presentation of findings.^51^

## Results

Three themes were identified from the data as shown in Table 1. They included perceptions of using DMH services, the perceived barriers to utilization, and suggestions on enhancement of DMH for YP in low-resource settings. There was a lot of discussion on the perceived barriers to the utilization of DMH across all FGDs.

**Table 1.** Themes.

Theme	Sub-theme	Key findings
1. Perceptions of using DMH services	Awareness of DMH	*There is also like the real-time consultation with someone, a mental health specialist or a therapist where you do not necessarily have to be present, like physically present with them, you are having a real-time consultation with a psychiatrist or psychologist.* (FGD 1) *I know that Makerere University has an online engagement under the counselling and guidance centre. I forget the name of the app but there is an app where you can go and text, I need a counsellor and immediately someone will reach out to you, and you can be able to talk through it.* (FGD 2)
2. Perceived barriers to utilization of DMH	Lack of internet-enabled gadgets and limited internet connectivity Inequitable access to DMHI Negative perception of mental health and the fear of stigma App-related challenges Mental health is neglected in Uganda Privacy and confidentiality concerns	*Most of the [DMH] services we have in Uganda, maybe in rehabilitation places are in town, in Kampala city but we don’t have these places upcountry…* (FGD 1) *Maybe the other challenge is ignorance about the existence of these platforms. Many young* [people] *don’t know that these platforms are there. So,* [when] *they get problems, they don’t know where to run to and they end up dying with their own problems.* (FGD 1) *We have been brought up as Africans, we believe these things* [mental illness] *don’t exist.* (FGD 2)
3. Enhancement of the utilization of DMH by YP in low-resource settings	Public engagement and sensitization Increased government funding on mental health services and regulation of *DMH* Enhanced training of health workers in mental health Establishing a toll-free line DMH apps should be affordable	*So, I talked about the toll-free system may be in order to scale up the involvement or the exposure to [digital mental health]. We can have toll free systems where someone can call, you don’t require credit or airtime and you get a professional to talk to you or someone who can help you with your condition.* (FGD 3) *So, the mere fact that we can increase education to all the people that really these things are happening, and you or the other person can be suffering from such a condition, would be a very big plus. And then informing the people about what is being done to solve these problems. After telling them that you can have such a problem, what can we do to solve it? So, we have this app, or we have this and this. But if I don't know of anything that is going to help me, definitely don't look out for anything.* (FGD 6)

### Theme 1: perceptions of using DMH services

#### Awareness of DMH

Overall, most respondents were aware of the availability of DMH technologies and some of them had used them before. They defined DMH as access to mental health solutions using modern online technologies. Respondents mentioned that these digital technologies can be used on ordinary mobile phones or smartphones for voice calls, text messaging, browsing mental health-related websites, and accessing mobile apps. Respondents mentioned a range of DMH solutions they had either used or had heard about, and these included but were not limited to mobile apps, zoom, podcasts, blogs, YouTube videos or channels, *edutainment*, and social media.

I know of three platforms; the first is Zoom, where people share their stories about mental health; their experiences are basically to help others or one or two people who can be inspired by that. Then the next is podcasts. There are people who do podcasts and it's like episodes about mental health, for example, coping with stress, coping with that but it is basically peer mentoring. And then the other is blogs where people write articles or pieces, basically about their experiences or what they know about mental health and so they share links, and then people can get access to those kinds of material. (FGD 1)

Several mental health-related apps were also mentioned including ‘*Unicare*’, an open-access professional counselling app for Makerere University students and employees, *Moodfit*, *TikTok*, *WHOVA*, *Reddit*, *Talkspace*, *Quora*, and various meditation apps. However, respondents expressed a preference for apps that have a human touch and can consistently be used over time as illustrated in the following quotes.

When I am to use an application, I would need an application that has AI [Artificial Intelligence] in it. That means, at some point, I shift from AI to chatting with a real person. It can be a person I don’t know and an application that maybe has a social aspect whereby I can access a friend through the same app, something like that so that I can be able to get comprehensive…. (FGD 1)

And then also, if I am using a digital mental health intervention, I would consider consistency a lot because for someone to have the mental health problems solved, it must be over a specific period of time which is usually very long. So, if I'm to use an application, I would like this application to sort of give me comprehensive help and help that is consistent. (FGD 1)

Respondents across all FGDs opined that DMH has helped extend mental healthcare to YP who would otherwise have challenges in accessing such services. They however indicated that it was difficult to figure out the right app to use for specific mental health problems.

…it's really very hard to know exactly which application to use, and which one is better in terms of linkage maybe to care and getting the services that I might want. (FGD 4)

### Theme 2: perceived barriers to utilization of DMH

#### Lack of internet-enabled gadgets and limited internet connectivity

The hindrances to access to digital mental health interventions (DMHIs) mentioned across all FGDs were limited access to internet-enabled electronic gadgets and expensive and unreliable internet connectivity. Respondents opined that most DMH solutions can only be accessed on internet-enabled gadgets however, many YP do not have smartphones, laptops, or other internet-enabled gadgets. Some respondents indicated that some YP have smartphones but do not know how to use them. They appreciated the efforts taken by the government and telecom companies in expanding internet services in the country. However, they noted that many rural areas still have limited internet connectivity.

Then, also accessibility is the network issue. Whereas we have a high percentage of internet penetration in Uganda, we keep on increasing but in terms of the network in most of the places especially rural settings, it is very poor. It is a struggle to get a network in these places. (FGD 5)

Some respondents surmised that this creates inequality in the provision of mental health services in the country. One respondent narrated his experience:

Yes, there was a time I was in Karamoja [a remote region in northeast Uganda], and then a colleague had a serious mental breakdown. Because there are no services on that side … So, we tried to access a mental health intervention from the internet, and we failed….. (FGD 6)

#### Inequitable access to DMH interventions

Respondents expressed concerns about the inequitable access to DMHIs in Uganda. They pointed out that most mental healthcare and rehabilitation services are only available in urban areas. They also surmised that most DMH apps are only accessible to the literate, which they felt was unfair because many YP stay in rural areas, and most of them can neither read nor write. Some respondents suggested that these factors should be considered during digital app development.

They should develop this app or policies or something that is in line with the level of literacy in the country not only to cater for a certain group of the literate but then leave out the illiterate because the target has not been achieved over time. The literacy targets. So, we know most of the people are still illiterate. (FGD 6)

Another hindrance to access to DMH interventions cited is the high cost of DMH apps. Some respondents noted that several online DMH resources require payment and most YP cannot afford them. Furthermore, it was noted that most apps require credit or debit cards to effect payment, yet most YP do not possess them.

In addition to what …  has said, in our Ugandan setting, not everyone has those cards, the credit cards. So sometimes it is just hard to incorporate it into our system, the dollar and how much to pay. (FGD 1)

#### Negative perception of mental illness and the fear of stigma

Mental health disorders and DMH were perceived as foreign to Africa. Some respondents felt that YP have negative attitudes towards mental health services and perceived therapy as a ‘*white people*’ problem. One respondent contended that all the digital health apps he had come across were in English, as such, he considered DMH as tailor-made for non-Africans, as illustrated below.

I also agree with participant […] about the stigma, that these things are for the whites, and even the interventions like when you Google it is not something African that will come up first let alone something in Uganda that will come up first in your search engine. And even the apps, I have not seen any app that is African, that has anything to do with mental health. (FGD 6)

Many respondents across all FGDs said YP are less likely to open up about mental health problems because of the stigma, negative perceptions, myths, and misconceptions associated with mental illness. They mentioned that mental illness is often attributed to witchcraft and evil spirits. They also noted that many people in society do not know the difference between mental health and mental illness. Respondents felt that these negative perceptions not only hinder people from seeking professional assistance but also potentially influence the use of DMHIs.

…but unless people start embracing that mental health is not actually running mad, because when you talk about mental health, whatever might come into someone's mind is, ‘Now this one has gone mad’. That is where the problem is. So, when someone thinks, okay, I'm not mad, I don't have any mental health problem, so I don't need to use this app or any other. (FGD 4)

So, my idea is about perception. Most people think mental health is caused by witchcraft. So, we first need to sensitize our people to know about mental health and change our mindset because in the village the people tend to go to traditional solutions, instead of health and scientific conditions. Most people tend to think maybe someone has bewitched them, so they end up leaving someone with a mental health issue, and the condition worsens. (FGD 5)

One respondent added that people perceive seeking help for a mental health problem as a sign of weakness rather than a strength.

I think the biggest problem is the setting and the image the setting has painted of mental health. Generally, people know that it is bad to have such an experience of mental health [disorder], and the community has in a way taught people that seeking assistance is a sign of weakness. So, most people will actually know that I am depressed, I have anxiety, I am not okay, but I will not look for help because I want to look strong. Yeah, it is part of a community problem. (FGD 1)

It was noted that some YP have access to DMH apps but do not share them, let alone use them, because of the judgmental nature of the community and fear of being deemed ‘mad’ as illustrated in this quote:

The other big issue is the stigma that comes with using such interfaces. So, it is quite not easy to tell a friend that I am having this, and I am using this and this to go about this. So, wherever there is stigma for something, which means its use is going to be limited. People will not even accept in the first place that I need help. So even if there is any way we can find a solution to the stigma that comes with it, we can increase its accessibility and use. (FGD 6)

#### App-related challenges

Respondents mentioned several app-related factors that could affect access to DMH solutions. One respondent pointed out that some apps are not available in mobile phone ‘play stores’ while others are tailor-made for different phone versions, and this may limit their availability. Several respondents also felt that some apps are boring, have unattractive user interfaces, are difficult to log-in, and are annoyingly slow. They contended that some apps require lots of bureaucratic steps to access them. Another respondent pointed out that some apps may not be appropriate for certain mental health conditions.

Sometimes you find that things are not tailored at all for your exact problem. There are many apps with general advice on how to handle a situation. You realize that everyone's situation is unique. So, in many cases, there are those apps including […], you are reading something that is tailored to someone else's problems, or it is [so] broad that it does not feed directly into your problem. So, you end up not getting help. (FGD 1)

Some respondents noted that most apps are only available in English and lack local context; as such, they may not be appropriate.

For accessibility sometimes you just stop at English, like all of these things we are talking about, we are doing them at the level of English, and you forget that not everybody speaks English or not everybody has a good command of English. It would be good to think globally but in the local context. (FGD 1)

… Yes, because I feel like many are failing to solve our problems reason being, [that] they are made by people in other places, experiencing various challenges and having a [different] understanding of life. (FGD 5)

#### Mental health is neglected in Uganda

Some respondents observed that mental health in Uganda is neglected because of the little attention and investment by the government. Several respondents felt that most YP in Uganda have limited knowledge about the availability of DMH services. They noted that many YP do not even know how to utilize DMH services. It was noted that some YP may be well conversant with DMH but find difficulty in figuring out which interventions are best suited to their mental health problems.

I will also say that mental health is not well developed […] in Uganda to a wide extent. But even the issue of the development of these apps or digital mental health will least be thought about. It is not so much developed in the country. It is taken as a minority, sometimes by the population or even the medical field or even the psychological field. It is not so developed that is why sometimes we don’t even know that we are having a mental breakdown because it is not so much a point in Uganda. (FGD 6)

#### Privacy and confidentiality concerns

Many apps and websites ask for a lot of information, including sensitive personal data. Some respondents reported feeling vulnerable whenever they use online mental health resources because they are uncertain of who is responding to their problems, and the security of their personal information.

I am also thinking, about what would stop me, why I would choose to talk to a person rather than go for an app is about privacy issues. I don't know who is behind the app, who is responding to my mental things, [….] So, if I know that I talked to maybe [….] about my issues, she will give me a direct answer there, and then I trust her. I don't know someone who is behind the app, who is responding and such things. (FGD 3)

### Theme 3: enhancement of the utilization of DMH by YP in low-resource settings

#### Public engagement and sensitization

Respondents offered several suggestions on how to improve access to DMH services in Uganda. They emphasized the need for public engagement and sensitization campaigns to create awareness of mental health and the availability of DMH services. They said that this could be done through regular advertisement of DMH services using social media and mass media. Furthermore, they suggested that these campaigns should be multi-sectoral and should involve various stakeholders including policymakers, researchers, academicians, community leaders, social media influencers, and members of the public. They also recommended research to understand community perceptions of mental health and the causes of mental health problems.

I think first of all the government should think of implementing a certain community engagement project. Maybe they should work with professional researchers, academicians and community leaders and do community engagement research … . If the government is to come in, and they are to push for digital mental health interventions, without actually going down to the grassroots, getting to know what people think about mental health and what they perceive as mental health problems. (FGD 1)

Another participant emphasized the importance of obtaining baseline information before developing DMH interventions:

We need to recognize that the mental health problem in Uganda is a structural problem. The organizers should at least sit down and do a root-cause analysis. Why are we here and how did we get here? And when we have done that, another thing we should think about is infiltration. People need to learn. There needs to be a lot of learning before we can introduce this [DMH]. (FGD 1)

Some respondents felt that the information collected should be integrated into the design of DMH apps and ensure that people's preferences are duly considered.

I think it's all about data collection. Is the information there? Do we have the right figures about mental health, about the different mental health challenges that exist to base on to make a given decision? To start with, I think the drive would be about getting the right data. You would want to know, okay, if there are mental problems and what would be the causes, I mean, right from down where you are suggesting. We need to have data about these challenges. I think before we think about anything, any solution, you need to have the data. (FGD 4)

#### Increased government funding for mental health services and regulation of *DMH*

Respondents recommended prioritization and increased government funding of mental health services and the regulation of digital health technologies. They also opined that the government should take on the responsibility of ensuring that DMH services are available to all Ugandans. They, however, contended that DMH interventions should be well-regulated to ensure that they are appropriate for YP and are safe to use. They suggested the development of frameworks to regulate the development and use of digital health applications. They advised that such regulatory frameworks should be developed systematically based on empirical evidence and stakeholder involvement.

We cannot just come up with the policy, the first step is identifying the problem. And when you identify the problem, you must go through a down-bottom approach. This [involves] going to the people, not you are sitting and coming down. Then after you have done the research, you put it on the agenda. This is where people analyze, weigh, and after putting it on the agenda, bring the actors. Who should we put? Government, NGOs? After that, look at the resources then we implement. (FGD 5)

They also emphasized the need for measures to sanction offenders who abuse or misuse digital health technologies.

Talking about policy still, privacy, and confidentiality should be very paramount when discussing policies in digital mental health. There should be legal aspects to how to do this whenever they are broken as well. (FGD 1)

#### Enhanced training of health workers in mental health

Some respondents mentioned that there are few mental health professionals in the country and felt that mental health is not given the attention it deserves in training curricula. One respondent recommended basic mental health training for all health care providers in mental health, right from the village health teams, that are at the bottom of the health system in Uganda. Village health teams work within the community and are perceived as more accessible; therefore, training them would improve access to mental health services.

So going back to her point of training these VHTs [village health teams] about mental health, because people already know them, and there are already existent systems to compensate these people, or their work and even any other resources they may need and help people at a community level. (FGD 1)

#### Establishing toll-free lines

Respondents suggested that the government should negotiate with telecom companies to establish toll-free lines that people with mental health problems can call and receive help. They indicated that this was important for people without smartphones and those in areas with poor internet connectivity. They asserted that the people who answer the phone calls should receive special training and should act professionally since they will be dealing with people with mental health problems.

I think the government should also train people who will be in place to actually attend to the individuals who will be coming in for mental health services. Because if at all we put in place toll-free lines, but then when they call one is actually at the other end to receive […]. And we need these people to be taught how to be patient. Some people know how to practice it, but some people need to be taught because if you are dealing with someone having a mental health issue, you need to be patient because in most cases you have actually never experienced what they are going through. So, you may look at it as a waste of time, but you are forgetting they need that time to process what it is they are going to air out to you. (FGD 1)

#### DMH apps should be affordable

Across all FGDs respondents suggested that the government reduce taxes to make smartphones more affordable. They also asserted that DMH apps should be accessible, completely free, and easy to use.

About accessibility, what I wanted to say is that it should be completely free because if there is any cost incurred whether for data or subscription, trust me people won’t go for that app. You are stressed and people are making you pay for data. No. No way. So, it should just be free completely. That is all Ugandans want. Free things. (FGD 5)

## Discussion

Overall, most respondents had a positive attitude towards DMH and some of them had used these interventions before. Several perceived barriers to the adoption of DMH were identified and possible suggestions on the enhancement of access to DMH interventions were proposed.

This study was conducted during the COVID-19 pandemic, at a time when many people faced mental health challenges.^21,29^ Many YP suffered increased depression, anxiety, and psychological distress, and lacked access to mental health care.^52^ At the time, there was increased demand and utilization for DMH solutions globally, particularly among adolescents and YP.^53,54^ In Uganda, there was increased utilization of digital health interventions including mental health support services such as ‘tele-counselling’ and ‘telepsychiatry’.^55^ DMH interventions have been reported to be beneficial to university students,^56^ and we believe this may have contributed to the positive attitude our respondents had towards DMH. However, despite the potential benefits, DMH technologies seem to raise technical, scientific, ethical, and regulatory challenges. Addressing these challenges is paramount to the successful implementation of digital health in these settings.

Almost all respondents owned smartphones and had prior exposure to DMH apps. This being a relatively, new concept among YP in Uganda, we aimed to gather meaningful information from respondents who were digitally literate. Most digital health apps are only available on smartphones, thus the assumption that individuals who owned a smartphone had a level of digital literacy. We also assumed that smartphone owners were more likely to have prior exposure to DMH apps and other online health solutions. We believe we would not have obtained meaningful information if a greater proportion of our respondents did not own smartphones.

Some of our respondents had used DMH apps before and gave examples of apps they were either aware of or had used before, including an institution-owned app at one of the universities in the country. However, they felt that most YP in the country are unaware of the availability of DMH services. They also felt that many YP do not know how to use DMH apps, let alone how to access them. This finding resonates well with a recent study from South Africa that reported a positive attitude towards the use of digital apps in improving mental health literacy by 92% of respondents. However, only 50% in that study had come across mental health apps and none had used them before.^38^ Our respondents’ low awareness and utilization of DMH to the lack of smartphones and other internet-enabled gadgets, widespread digital illiteracy, and unreliable internet access. Furthermore, they indicated that most DMH interventions are only available in English, lack local context, and may not be appropriate for most YP in Uganda. All of these challenges have been reported in the literature and are not unique to our setting.^30,38,40,57,58^ Inequitable access and low digital literacy may deprive YP of much-needed mental health care solutions.^59^ As our findings suggest, there are concerns about the *digital divide*, a phenomenon where technology is not equally available due to economic, social, and cultural inequalities, and low technological proficiency.^60–62^ According to *Statista*,^63^ 93% of individuals in high-income countries have internet access compared to 27% in low-income countries, with Eastern Africa having the lowest internet rate (26.8%). Notably, YP are the most active users of the internet.^63^ Closing the digital divide requires multi-sectoral concerted efforts from educators, parents, and the YP themselves. Several approaches have been used to enhance digital literacy including encouraging self-education, peer-learning, parental involvement and setting boundaries for use of electronic gadgets, fostering responsible online behaviour, and initiating educational programmes in schools and communities.^64,65^ These are actionable approaches that could be easily adopted in Uganda to enhance both digital literacy and DMH.

Another perceived hindrance to the use of DMH interventions mentioned in this study is the negative attitude and stigma associated with mental illness. Stigma has detrimental effects on those suffering from mental illness and poses a serious challenge to the health-seeking behaviour, treatment, and prevention of mental illness.^66^ Globally, mental illness is highly stigmatized.^67^ Similarly, in Uganda, mental health-related stigma is highly prevalent and is a common response to mental illness in many communities.^68^ Internalized-stigma or self-stigma defined as ‘the shame and expectation of discrimination that prevents people from talking about their experiences and stops them seeking help’,^69^ is common in mental illness and can lead to shame, low-self-esteem, and social isolation, a feeling of hopelessness, relational difficulties, increased psychiatric symptoms, and poor adherence to treatment.^70–72^ Generally, stigma is also associated with poor quality of life and adverse health outcomes. Therefore, it is important to come up with interventions that seek to reduce stigma and discrimination, and increase access to treatment.

Several stigma-reduction methods have been employed in the literature. For example, community-led theatre-based de-stigmatization of mental illness has been successfully been used to reduce community stigma and discrimination^73^; STRETCH (Stigma Reduction to Trigger Change for Children)^74^; and digital technologies.^41,75^ Digital technologies have been successfully used in the treatment of individuals with mental illness who may be hesitant to seek care due to stigma.^76,77^ As our findings suggest, one way of reducing stigma is through creating awareness of mental health. Digital technology can help achieve this through addressing inaccurate misinformation and negative portrayal of mental illness; providing truthful information to the public to enable better understanding of mental illness and improving attitudes towards people with mental disorders; creating opportunities for peer connection where people with mental disorders link up and support each other; and encouraging people living with mental illness to openly share with the public about their challenges.^78^ Therefore, there is a need to improve the utilization of DMH interventions because they are key in improving mental health literacy,^79^ reducing stigma,^77,80^ and increasing willingness to seek help.^78,81^ However, although digital technologies present opportunities to address mental health stigma,^78^ they could also exacerbate it, especially when sensitive personal information is exposed.^66^

Our respondents expressed major concerns about the safety of the personal information gathered by digital health applications. They expressed wary of exposure of people's identities or information being traced back to an individual in case of any breach in confidentiality. Privacy, confidentiality, and data security are the most frequently mentioned risks of using DMH technologies,^66,82^ particularly when confidential information is shared with third parties.^83–86^ It has also been shown that some mental health apps often lack crucial privacy policy provisions and exhibit inadequacies in security and privacy best practices.^87^ The prospect of loss of privacy and security vulnerabilities may decrease the acceptability of DMH.^88,89^ Similarly, mistrust and the feeling that technologies collect a lot of information may be a barrier to adoption, and reduce the effectiveness of DMH.^66,90^ Therefore, there is a need for robust security protocols to ensure that people's data is protected as prescribed by data protection and privacy laws. There is also a need for specific regulations to protect data from personal devices and specifically, to establish standards on data storage and transmission that ensure that people's mental health data is only used for its intended purposes.^33^ Our respondents recommended the instituting of measures to sanction offenders who abuse or misuse digital health technologies.

There has been an increase in the utilization of digital health technologies in Uganda since the COVID-19 pandemic,^55^ however, there is poor regulation. Many health apps are marketed with almost no checks or regulatory approval. Our respondents rightly recommended the regulation and accreditation of DMH services to ensure that they are appropriate for YP and are safe to use. It is important to note that in Uganda, there are several regulatory frameworks for eHealth; however, none of them is specific or robust enough to regulate DMH. Digital health in Uganda is governed by the national e-health policy.^91^ However, this policy acknowledges the lack of standards and national oversight for local eHealth innovation. Inadequate regulation of DMH is a global challenge that is not unique to LMICs only.^92^ For example, only a few of the DMH tools currently being used in the USA have been clinically tested and authorized by the United States Federal Drug Authority.^92^ It is important to note that the International Medical Device Regulators’ Forum, a global voluntary group of medical device regulators has developed detailed guidance on definitions, a framework for risk categorization, quality management, and the clinical evaluation of digital medical devices^93–95^ that can be used for reference as countries develop their own regulatory frameworks for digital health. For best practices, digital technologies should be integrated with traditional care,^33,96^ and health professionals should receive training and education on DMH. They should also be involved in setting forth appropriate models for integrating DMH in clinical care.

Our findings suggest that YP are often spoilt for choice because of the many apps available on the market. They indicated that they need guidance on what apps to use. It is well established that some DMH apps are not only ineffective, unsafe, and difficult to use, but may not meet users’ privacy and security expectations.^97–99^ Therefore, there is a need for consumer protection. Consumers need basic information to help them choose an app that is best suited for their mental health problems. Wykes and Schueller proposed the Transparency for Trust (T4T) principles with the goal of operationalizing efforts for better regulation and backing up digital health products with empirical data.^100^ These principles include privacy and data security, development characteristics, feasibility data, and benefits. The T4T principles propose a set of questions that could provide information to users during app downloading and allow them to make an informed decision.^100^ This is important because DMH technologies can potentially increase the autonomy and sense of empowerment of YP, but they also increase the risk of diminishing patient autonomy by accentuating the risk of digital addiction and manipulation.^66^ The use of health apps may also delay treatments that could be more beneficial thus, the opportunity cost of using the app should be evaluated. Therefore, DMH technologies should be well-regulated and used with caution, preferably on the recommendation of a health professional.

### Strengths and weaknesses

The main strength of the study was the involvement of students from diverse academic backgrounds. To the best of the authors’ knowledge, this is the first study to delve into DMH in Uganda, and we needed to recruit individuals we felt had an idea of digital health. We used FGDs because the phenomenon under study was relatively new to most YP. We also wanted to quickly collect in-depth information cost-effectively because of the financial hardships during the pandemic. The focus group dynamic stimulated debate and respondents to build on one another's responses and generate idea that they might not have fathomed out in individual interviews. We also aimed to cost-effectively obtain quick feedback from a relatively diverse group of people.

The main weakness of our study was the involvement of only university students. We aimed at a study population that would help us obtain meaningful data on YP's perceptions of DMH because of the low digital literacy and novelty of DMH in Uganda. However, we tried to address this by recruiting participants from nine private and public hospitals, and from diverse academic programmes. By so doing, we obtained opinions and identified common themes and perceptions across various groups of YP. We may have missed out on important perceptions of individuals with a low level of education, and those from rural areas. However, we believe our findings provide a glimpse of the perceptions of YP in the country since they resonate well with the literature.

We also acknowledge potential sources of bias from the moderators, participants, or the group. As the moderators, we were aware that when interviewing YP we needed to try and remain neutral, setting aside our views, and listening from the respondents’ perspective. It was however difficult for us to be totally objective and not relate to our experiences because of our interest in the ethics of mental health and enhancing access to mental health services to YP in resource-limited settings. We tried to overcome this through protocol training and ensuring that the FGDs are moderated by an experienced team that was relatively unknown to the majority of participants. Participants may have had different expectations that could have influenced their responses. They also could have withheld their genuine perceptions and instead offered responses they believed were socially desirable. However, we tried to create a comfortable and friendly atmosphere, encouraged active participation, and iterative discussions, and gave all participants a chance to voice their opinions. Last, the use of a snowballing approach could have led to a biased sample because participants tend to recommend individuals with common preferences and is greatly influenced by social networks. This study was conducted during the COVID-19 pandemic shortly after travel restrictions were lifted and all educational institutions in the country were closed. As such, participant mobilization using probability sampling methods was practically impossible, and that is why we opted for a snowballing technique. Despite these limitations, we hope the findings of this study will stimulate more inclusive research on DMH.

## Conclusion

Awareness of DMH among participants was high; however, there was a general feeling that most YP are not informed about the availability of digital solutions to mental healthcare. DMH was deemed important in extending much-needed healthcare to YP in a country where mental health services are less prioritized, poorly funded, and not readily available. The findings suggest that equitable access to DMH interventions is influenced by the lack of internet-enabled gadgets, unreliable internet connectivity, stigma fears, privacy and confidentiality concerns, and app-related challenges. YP expressed a preference for DMH apps that are cheap, user-friendly, can be used consistently over time, and provide real-time consultation where a person in need of mental health care can interact with a mental health specialist without necessarily having him/her physically present. DMH interventions were also seen as less stigmatizing than traditional care. Access and utilization of DMH interventions can be enhanced through public engagement, creating awareness of the availability of DMH interventions, enhanced training to increase the number of mental health professionals, and access to affordable DMH interventions.

### Best practices

Our findings suggest the need for increasing public awareness of DMH. The thought of up-scaling DMH services in Uganda is appealing however, we posit that widespread adoption of DMH may exacerbate existing health inequities.^101^ Therefore, there is a need for long-term investment in digital health technologies aimed at tackling structural barriers that generate health disparities. This could be achieved through increased government investment in mental healthcare, clinician training, improving digital literacy, reliable internet connectivity, and developing context-specific regulatory frameworks to regulate the development and use of digital health interventions.^101^

There is also a need to evaluate the available DMH apps and the feasibility of making them freely available to the general population, particularly the YP. The government, together with other stakeholders should work towards enhancing the availability and usage of DMH interventions in the country. This could be achieved by ensuring digital health tools are affordable, easily accessible, culturally acceptable, and translated into the most spoken local languages to promote equity.

To maximize the potential of DMH, there is a need to close the digital divide and improve digital literacy. This may require active inter-sectoral collaboration involving governments, public sector, educational institutions, organizations, and communities. Further, digital education should be emphasized and included in national curriculums at all levels.^65^ Regional experts should be trained and deployed in rural and hard-to-reach area to act as champions for promoting digital education.

### Research agenda

DMH is relatively new in much of sub-Saharan Africa, and a lot needs to be done if it is to be embraced. Research should be conducted to understand the perceptions and preferences of local stakeholders including the public, clinicians, researchers, policymakers, programmers and app developers, and software engineers to mention a few. This information will not only help policymakers but also guide local innovators to develop context-specific digital tools to cater to the YP from diverse cultural backgrounds. Good developmental practices require the involvement of all stakeholders (including end-users) as well as using evidence-based guidance in all stages of app development. For enhanced utilization, app developers should design systems that are safe, trustworthy, and per the values and preferences of the end user.^33^ Studies should also be conducted to determine usability and user experience, user engagement, and whether there are any safety concerns regarding DMH.

## Abbreviations

COVID-19: SARS-CoV-2 virus
DMH: Digital mental health
DMHI: Digital mental health intervention
ECR: Early carrier researcher
EMDIYA: Ethics for Mental Health Digital Innovations for Young People in Africa
FGD: Focus group discussion
LMIC: Low and middle-income countries
STRETCH: Stigma Reduction to Trigger Change for Children
VHT: Village health teams
YP: Young people
YPAG: Young people's advisory group
